# Combination of retinoids and narrow-band ultraviolet B inhibits matrix metalloproteinase 13 expression in HaCaT keratinocytes and a mouse model of psoriasis

**DOI:** 10.1038/s41598-021-92599-w

**Published:** 2021-06-25

**Authors:** Chan Xi, Chuanxi Xiong, Huiping Wang, Yuanjun Liu, Suju Luo

**Affiliations:** Department of Dermatology, Tianjin Medical University General Hospital, 154 Anshan Dao, Tianjin, 300052 China

**Keywords:** Skin diseases, Medical research

## Abstract

Matrix metalloproteinase13 (MMP13) can be released by keratinocytes and fibroblasts and involved in the pathogenesis of skin disorders. Retinoic acid derivative drugs include tazarotene and acitretin. Tazarotene/acitretin and narrow-band ultraviolet B (NB-UVB) irradiation are common treatment options for psoriasis. However, their impact on MMP13 expression in the context of psoriasis has yet to be determined. The expression of MMP13 was analyzed in patients with psoriasis. The effects of tazarotene/acitretin and NB-UVB on MMP13 expression were also investigated in a mouse model of psoriasis. Human HaCaT keratinocytes were exposed to acitretin or NB-UVB and then assayed for cell proliferation and MMP13 expression levels. We showed that patients with psoriasis had increased levels of MMP13 protein in skin lesions and serum samples. Exposure to acitretin and NB-UVB irradiation alone or in combination led to reduction of cell proliferation and MMP13 expression in HaCaT cells. Consistently, tazarotene treatment or NB-UVB irradiation attenuated imiquimod-induced psoriasis-like dermatitis and decreased MMP13 expression in a mouse model. Based on these from HaCaT keratinocytes cells and animal experiments, we suggest that tazarotene/acitretin and NB-UVB irradiation can inhibit the expression of MMP13 in HaCaT keratinocytes and psoriasis mouse models. Blockade of MMP13 activity may have therapeutic potential in improving symptoms of psoriasis.

## Introduction

Psoriasis is a chronic recurrent inflammatory disease caused by abnormal proliferation and differentiation of keratinocytes^1–3^. Matrix metalloproteinases (MMPs) are structurally related zinc-dependent endopeptidases capable of degradation of extracellular matrix (ECM)^4,5^. Dysregulation of MMPs such as MMP2 and MMP9 is frequently detected in patients with psoriasis^6,7^, suggesting an involvement of MMPs in psoriasis. MMP13 shows the ability to regulate multiple pathological processes such as arthritis, infection, and tumor progression^5,8^. Since MMP13 can modulate keratinocyte migration and inflammatory response in murine skin wound models^9,10^, we hypothesized that MMP13 might play an important role in the pathogenesis of psoriasis.

Both topical and systemic vitamin A derivatives, are used in the treatment of psoriasis. Tazarotene is a topical agent and acitretin is a systemic retinoid^11^. Both are most effective in combination with other treatment modalities. Tazarotene and acitretin are selective retinoic acid receptor (RAR) agonists. They show the ability to regulate keratinocyte proliferation and differentiation and inhibit inflammation^11^. Narrow-band UVB (NB-UVB) that emits mostly 311/312 nm lights is commonly used in phototherapy^12^. NB-UVB has a more profound therapeutic efficacy for psoriasis than conventional UVB, with a shorter period of time and fewer adverse effects. NB-UVB irradiation can result in lymphocyte apoptosis and decrease the number of Langerhans cells^13,14^. Psoriasis resolves to a greater degree in patients treated with NB-UVB in combination with retinoic acid than in patients treated with either NB-UVB or retinoic acid alone^15–17^. Despite these findings, the mechanism underlying the therapeutic benefits in psoriasis is still unclear.

In this study, we determined the expression of MMP13 in patients with psoriasis and investigated the influence of tazarotene/acitretin and NB-UVB irradiation on the expression of MMP13 in imiquimod (IMQ)-induced mice and HaCaT cells.

## Materials and methods

### Tissue sample collection

Eighteen cases of skin lesions were collected from patients with psoriasis who were diagnosed in General Hospital of Tianjin Medical University between May 2019 and August 2019. Exclusion criteria were ongoing systemic anti-inflammatory treatment, other rheumatological diseases, other inflammatory dermatoses, pregnancy, intense UV exposure 2 weeks prior to study start, chronic infectious diseases, exposure to photosensitizing drugs, and age < 18 years. The control group included 10 skin samples obtained from patients undergoing cosmetic surgery in General Hospital of Tianjin Medical University. Blood samples were taken for preoperative, and serum samples were stored at − 80 °C. Skin biopsies were paraffin-embedded and subjected to immunohistochemistry.

### Animal experiments

Twenty-five male BALB/c mice (6–8 weeks old) were purchased from Beijing Viton LiHua Co., LTD (Beijing, China). Mice were maintained at 24–25 °C under a 12 h light-dark cycle with free access to food and water. The study was performed in accordance with the Chinese Council on Animal Care guideline for animal research and approved by the Animal Care and Use Committee of General Hospital of Tianjin Medical University (IRB-2021-DW-02). The study was carried out in compliance with the ARRIVE guidelines.

All the mice were randomly divided into 5 groups: control group, IMQ-induced group, IMQ + tazarotene group, IMQ + NB-UVB irradiation group, and IMQ + tazarotene + NB-UVB irradiation group. Control mice were untreated. The IMQ-induced group received 62.5 mg IMQ cream on the shaved back daily for 7 days to induce psoriasis-like skin inflammation^18^. IMQ + tazarotene, IMQ + NB-UVB, and IMQ + tazarotene + NB-UVB groups received the IMQ treatment daily for 7 days together with tazarotene treatment, 300 mJ/cm^2^ NB-UVB irradiation, and tazarotene plus NB-UVB irradiation, respectively. The 2 × 2 cm^2^ skin on the back of mice was selected as the observation area. At the end of the experiment, all mice were sacrificed. Blood was taken from the eyeball of mice. The dorsal skin sample of mice was paraffin-embedded and analyzed for MMP13 expression. The epidermal thickness was quantified using a computer-assisted image analyzer.

### Histology and immunohistochemistry

For histology, skin tissues were fixed in 4% buffered formalin, embedded in paraffin, and sectioned. Consecutive sections were stained with hematoxylin and eosin (H&E) and SafraninO. For immunohistochemistry, sections were deparaffinized and rehydrated. Endogenous peroxidase was blocked with 0.6% hydrogen peroxide in methanol. Sections were incubated with anti-MMP13 antibody overnight at 4 °C. An appropriate BrightVision peroxidase system (Immunologic) was used. The staining was visualized with diaminobenzidine.

### Enzyme-linked immunosorbent assay (ELISA) for MMP13

MMP13 concentrations in the serum samples and supernatants of cells were quantified using a commercially available MMP13 ELISA kit according to the protocols provided by the manufacturers (Cusabio Biotech Co., Ltd., Wuhan, China). All analyses were performed in triplicate. Optical densities were measured at 450 nm using a microplate reader.

### Cell culture and reagents

HaCaT cells were assigned to the following groups: control, 1 μM acitretin, 50 mJ/cm^2^ NB-UVB, 1 µM acitretin + 50 mJ/cm^2^ NB-UVB, 100 mJ/cm^2^ NB-UVB, and 1 µM acitretin + 100 mJ/cm^2^ NB-UVB. HaCaT cells at a density of 1 × 10^5^ cells/well were cultured in DMEM (Gibco, Grand Island, NY, USA) containing 10% FBS (Gibco), and 2 mM glutamine at 37 °C under a humidified atmosphere of 5% CO_2_. HaCaT cells at an 80% confluence were exposed to 1 µM acitretin (Chongqing Huapont Pharmaceuticals Co., Ltd., Chongqing, China) and/or 50–100 mJ/cm^2^ NB-UVB (312 nm UVB light source, Spectroline, USA). After irradiation, the cells were incubated for additional 24 h.

### Cell counting kit-8 (CCK-8) assay

Cell proliferation was analyzed using the CCK-8 assay (Solarbio Co., Ltd., Beijing, China). In brief, cells were plated in 96-well plates (1 × 10^3^ cells/well) and incubated for 24 h. The cells were then exposed to 1 µM acitretin and/or 50–100 mJ/cm^2^ NB-UVB for another 24 h. CCK-8 solution was added to each well. After incubation for 4 h, absorbance was measured at 450 nm.

### Quantitative real-time PCR analysis

Total RNA from cells were extracted using Trizol RNA isolation reagent (Thermos Fisher Scientific, Waltham, MA, USA) according to the manufacturer’s protocol. One microgram of total RNA was used for the synthesis of firststrand cDNA. Real-time PCR assays were carried out using AceQ Universal SYBR qPCR Master Mix. The PCR primers were used as follows: forward 5′-AACGCCAGACAAATGTGACC-3′ and reverse 5′-AAAACAGCTCCGCATCAACC-3′ for MMP13; forward 5′-GTCCACCGCAAATGCTTCTA-3′ and reverse 5′-TGCTGTCACCTTCACCGTTC-3′ for $\beta$-actin. The cycling conditions used were as follows: 95 °C for 5 min, followed by 40 cycles of denaturation at 95 °C for 10 s, annealing at 60 °C for 30 s, and extension at 72 °C for 15 s. The relative amount of MMP13 transcript was normalized by the amount of $\beta$-actin transcript. The results were analyzed using the formula 2$^{-\Delta \Delta CT}$.

### Western blot analysis

HaCaT cells were lysed in lysis buffer. The protein concentrations were measured by Bicinchoninic Acid (BCA) Protein Assay. Protein samples (20 µg) were separated by SDSPAGE (10%) and transferred onto polyvinylidene fluoride (PVDF) membrane. The membrane was blocked with 5% fat-free milk and incubated overnight at 4 °C with anti-MMP13 antibody (Abcam, Cambridge, UK). After washing, the membrane was then exposed to secondary antibodies coupled to horseradish peroxidase. Immunoreactivities were detected using ECL reagents (Biyuntian Biotechnology Co., LTD., shanghai, China). Protein bands were quantified using Image J software.

### Statistical analysis

All quantitative data represent the results of three independent experiments conducted in triplicate. Each value is expressed as the mean ± standard deviation (SD). The data were analyzed with the unpaired, two-tailed Student’s *t* test or one-way analysis of variance (ANOVA), using SPSS 23.0 and GraphPad Prism 5.0 software. Differences with *P* values of less than 0.05 were considered statistically significant.

### Ethics statement

All studies involving human patients were approved by the Medical Ethics Committee of the General Hospital of Tianjin Medical University (IRB2021-WZ-014). Participants gave their informed consent in writing, and the study was conducted according to the declaration of Helsinki.

## Results

### Upregulation of MMP13 in patients with psoriasis

**Figure 1:**
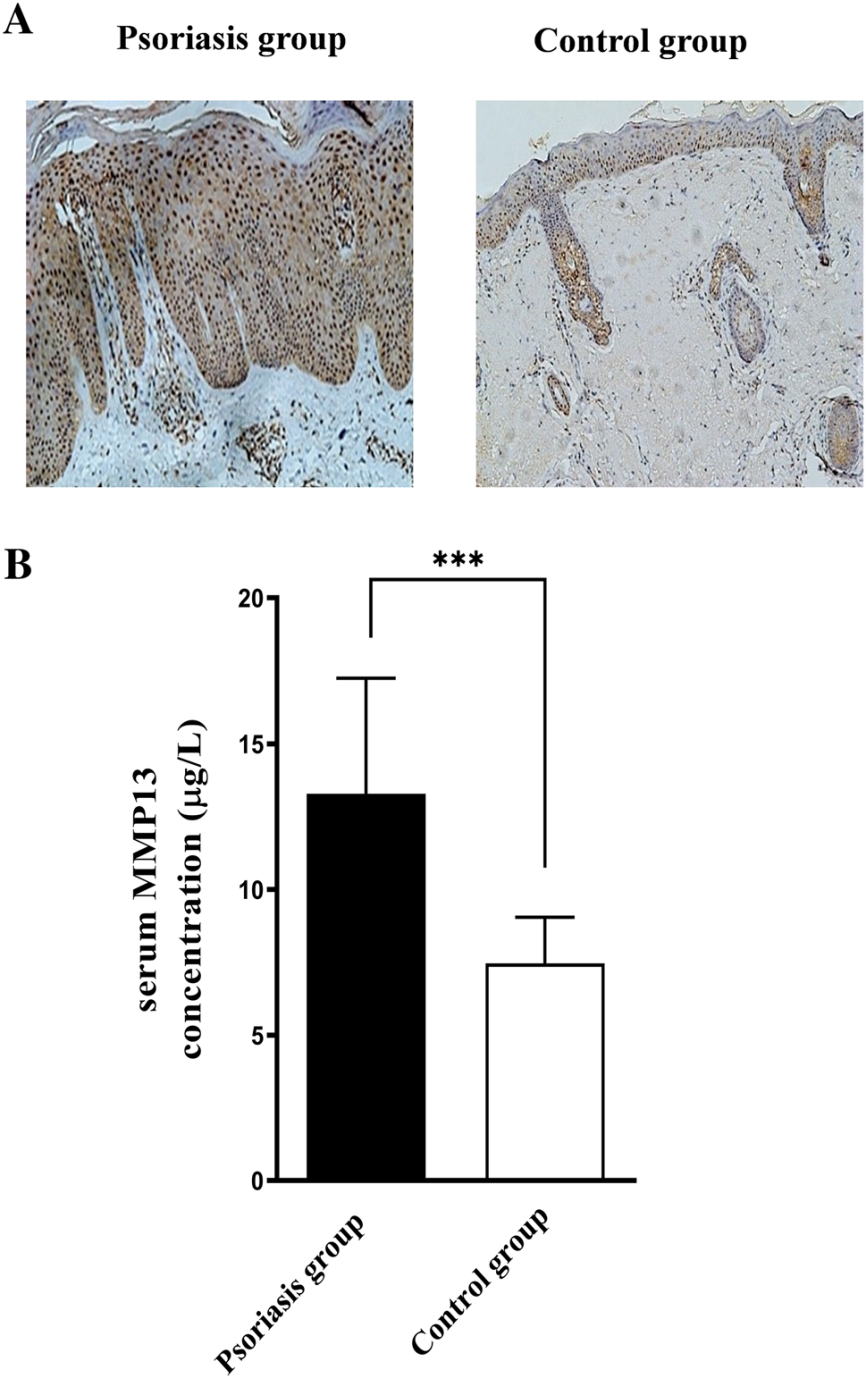
Expression of MMP13 in patients with psoriasis. (**A**) Immunostaining for MMP13 in psoriasis and normal skin tissues. Representative images of MMP13 staining are shown (×100). (**B**) Quantification of serum MMP13 levels in patients with psoriasis and control subjects. $^{*}P$ < 0.05 relative to control.

MMP13 staining was strongly positive in each layer of skin tissues in patients with psoriasis (Fig. 1A). In contrast, MMP13 staining was weak in the control group and mainly confined to the spinous and basal layers (Fig. 1A). Consistent with the upregulation of MMP13 in skin tissues, we showed that the serum MMP13 level in patients with psoriasis was significantly higher than that in the control group ($P < 0.001$; Fig. 1B).

### Effects of pharmacological treatment and NB-UVB irradiation on pathological changes in psoriatic mice

Compared to the control group, IMQ-induced mice showed increased skin thickness, hypertrophic spinous layer, and prolonged epidermal ridge (Table 1). IMQ-induced epidermal thickness was significantly attenuated by treatment with tazarotene or NB-UVB irradiation. The combination of tazarotene and NB-UVB irradiation led to a more profound improvement in skin thickness than each treatment alone.

**Table 1:** Quantification of skin thickness and measurement of serum MMP13 levels in mice after indicated treatments.

Group	Epidermal thickness (μm)	MMP13 concentration (μg/L)
Control	1.26 ± 0.04	185.76 ± 7.22
IMQ	7.93 ± 0.59^a^	215.98 ± 15.17^a^
IMQ + tazarotene	3.56 ± 0.37^b^	197.39 ± 3.92^b^
IMQ + NB-UVB	3.83 ± 0.39^b^	196.13 ± 11.76^b^
IMQ + tazarotene + NB-UVB	2.14 ± 0.34^b^	183.21 ± 14.99^b^
*P* value	< 0.001	< 0.001

^a^P < 0.05 relative to control group.

^b^P < 0.05 relative to IMQ group. Data represent the mean ± standard deviation. The data were analyzed using one-way ANOVA with Dunnett’s multiple test.

### Assessment of MMP13 expression in psoriatic mice

**Figure 2:**
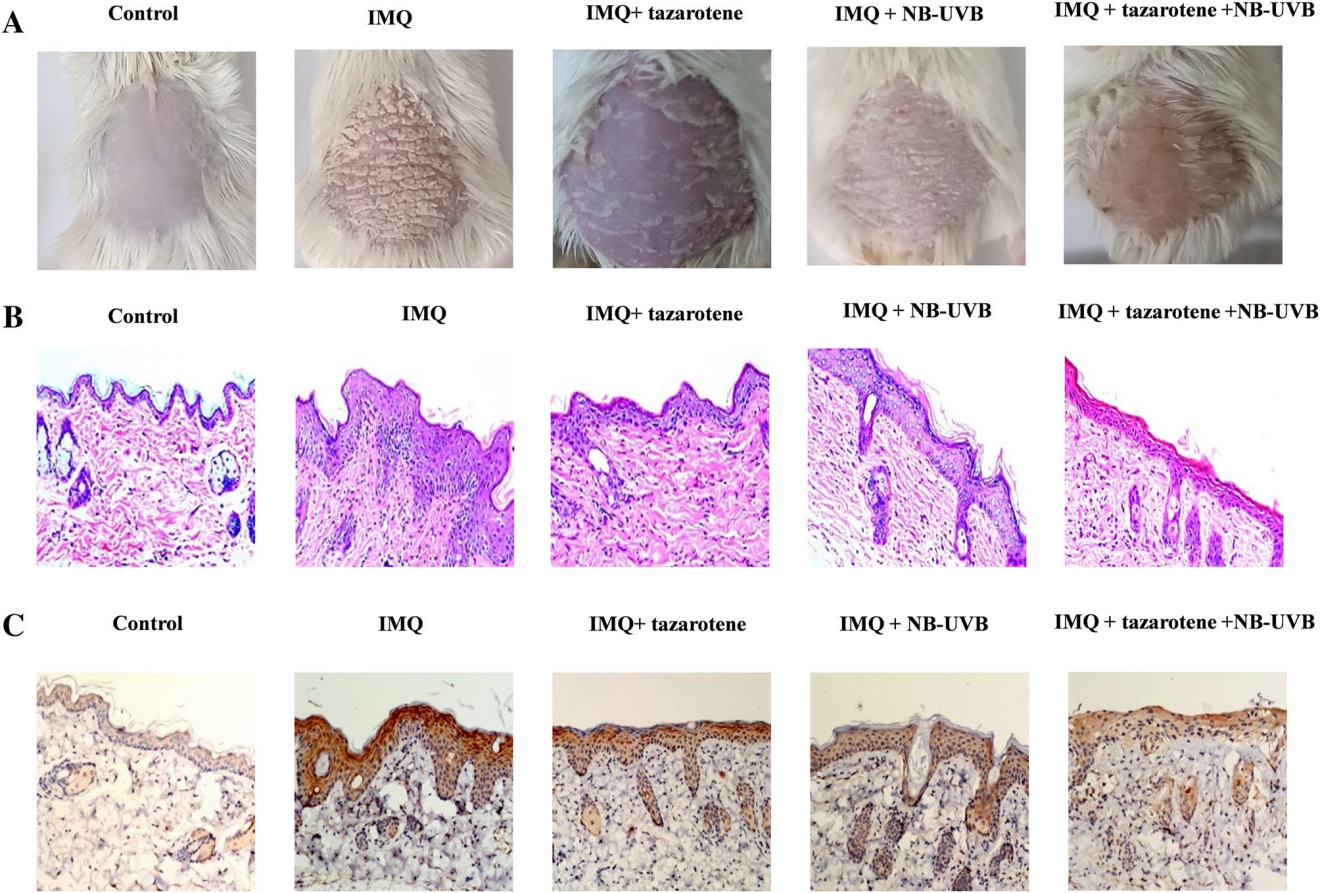
Effects of pharmacological treatment and NBUVB irradiation on pathological changes in psoriatic mice. (**A**) Representative photographs showing skin lesions of mice after indicated treatments. (**B**) Pathological changes in skin tissues from mice in treated as in (**A**). Representative HE staining images are shown (×200). (**C**) Histochemical analysis of MMP13 expression in skin tissues from mice with indicated treatments. Representative images of MMP13 staining are shown (×200).

Compared to control mice, skin lesions from IMQ-induced psoriatic mice had increased MMP13 expression (Fig. 2). Treatment with tazarotene or NB-UVB irradiation alone or in combination markedly prevented the IMQ-induced elevation of MMP13 in skin tissues (Fig. 2). Similarly, IMQ-induced mice had significantly greater levels of serum MMP13 than control mice. Serum MMP13 levels were lowered by treatment with tazarotene or NB-UVB irradiation (Table 1).

### Acitretin and NB-UVB irradiation inhibit cell proliferation and MMP13 expression in HaCaT cells

**Figure 3:**
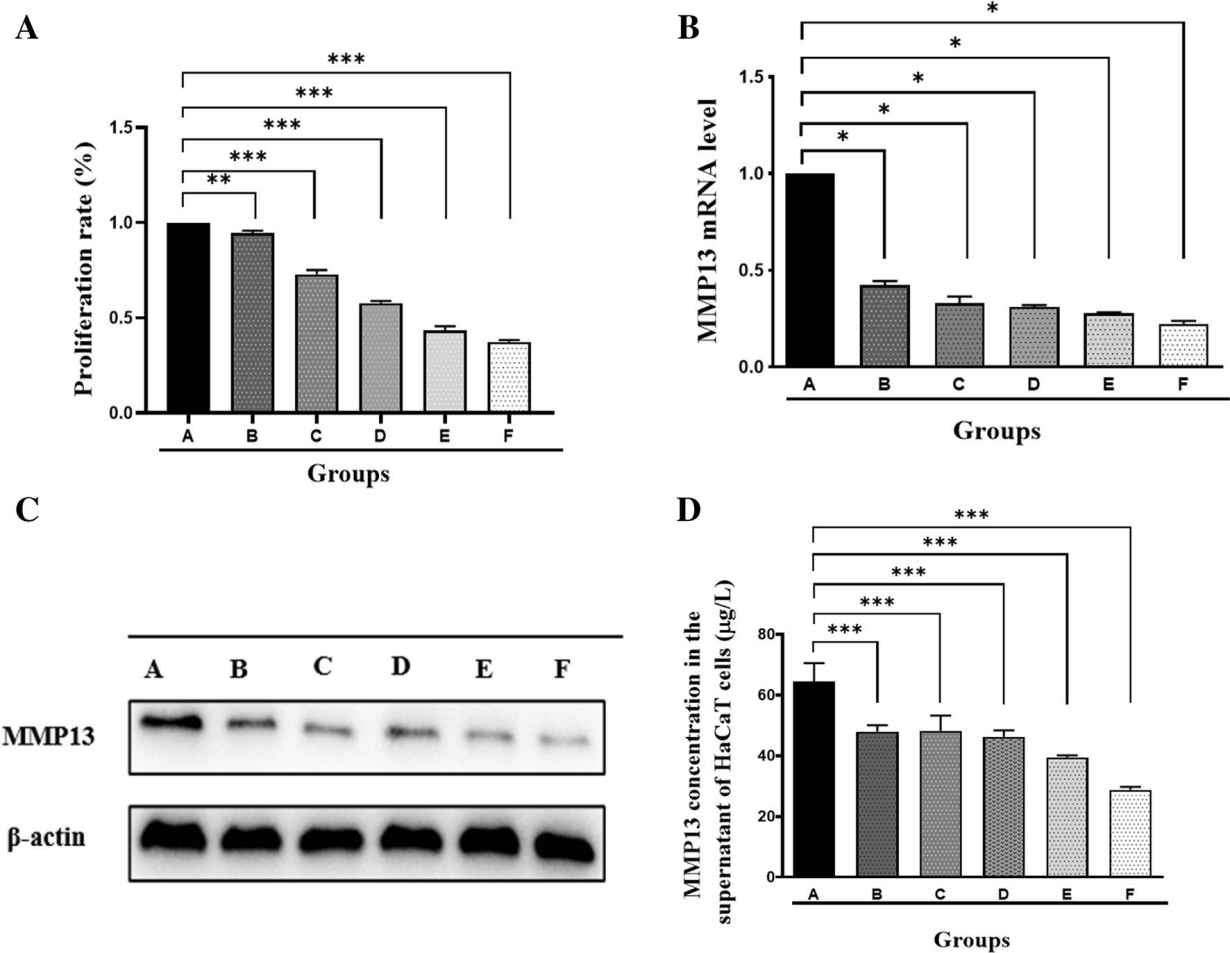
Effects of acitretin treatment or NB-UVB irradiation on HaCaT cells. Groups A–F: control, 1 µM acitretin, 50 mJ/cm^2^ NB-UVB, 1 µM acitretin + 50 mJ/cm^2^ NB-UVB, 100 mJ/cm^2^ NB-UVB, and 1 µM acitretin + 100 mJ/cm^2^ NB-UVB, respectively. (**A**) Analysis of HaCaT cell proliferation rate after indicated treatments. (**B**) Analysis of MMP13 mRNA levels in HaCaT cells treated as in (**A**). (**C**) Western blot analysis of MMP13 protein levels in HaCaT cells after indicated treatments. (**D**) Elisa analysis of MMP13 concentration in the supernatant of HaCaT cell. Data represent the mean ± standard deviation. $$^{*}P$$ < 0.05, $$^{**}P$$ < 0.01, $$^{***}P$$ < 0.001 relative to control group. The data were analyzed with one-way ANOVA with Dunnett’s multiple test.

Next, we investigated the effect of different treatments on HaCaT cells. As shown in Fig. 3A, the proliferation rate of HaCaT cells was reduced after treatment with acitretin or NB-UVB irradiation. Moreover, MMP13 expression was remarkably suppressed by acitretin or NB-UVB irradiation (Fig. 3B–D).

## Discussion

In this study, we showed that the levels of MMP13 in skin lesions and serum samples of patients with psoriasis were higher than those in the control group (Fig. 1). Combined treatment with acitretin and NB-UVB irradiation inhibited MMP13 expression in HaCaT keratinocytes (Fig. 3). Similarly, tazarotene and NB-UVB irradiation led to synergistic inhibition of MMP13 expression in an IMQ-induced psoriasis-like mouse model (Fig. 2 and Table 1).

MMPs mediate the degradation of different components of extracellular matrix and regulate cell adhesion, proliferation, migration, and differentiation. MMP13 can be released by keratinocytes and play a key role in psoriasis^19^. We found that acitretin and NB-UVB irradiation led to a downregulation of MMP13 at both the mRNA and protein levels, suggesting a transcriptional regulation. Given the suppression of MMP13 by acitretin or NB-UVB, we investigated the effect of these treatments on the proliferation of HaCaT keratinocytes. As expected, acitretin treatment and NB-UVB irradiation markedly inhibited the proliferation of HaCaT keratinocytes. The combination of acitretin and NB-UVB irradiation yielded synergistic effects on the proliferation of HaCaT keratinocytes (Fig. 3). In vivo studies confirmed that tazarotene and NB-UVB treatment suppressed MMP13 expression in skin lesions and blood samples of IMQ-induced mice (Fig. 2). Increased expression of MMP13 may promote the degradation of the ECM and thus affect the proliferation of keratinocytes^20,21^. High expression of MMP13 in psoriasis skin lesions may lead to changes in the microenvironment of keratinocytes and epidermal dynamics, consequently affecting the proliferation and differentiation of keratinocytes^22-24^. The changes of cells to cells and cells-matrix adhesion in the epidermis of psoriasis may be related to the abnormal increase of MMP13. Intriguingly, our data indicate that tazarotene treatment and NB-UVB irradiation inhibits MMP13 expression, which is accompanied by improvement in the psoriasis-like phenotype. Therefore, MMP13 may be a potential therapeutic target for psoriasis.

However, this study has several limitations. First, although we show that acitretin and NB-UVB irradiation can suppress MMP13 expression, the underlying molecular mechanism is still unclear. Our results reveal the reduction of both MMP13 mRNA and protein levels by acitretin and NB-UVB, indicating a regulation of MMP13 expression at a transcriptional level. Ongoing studies are conducted to explore the key transcriptional factor(s) mediating the regulation of MMP13 expression by acitretin and NB-UVB. Second, there is no direct evidence supporting that reduction of MMP13 expression is a causal factor for the improvement of psoriasis-like phenotype by tazarotene and NB-UVB. Third, we do not investigate the role of other MMPs in psoriasis pathogenesis.

## Conclusion

In summary, MMP13 expression is increased in skin lesions and serum samples of patients with psoriasis. Retinoid treatment plus NB-UVB effectively inhibits HaCaT cell proliferation and improves psoriasis-like phenotype in psoriatic mice. Retinoid and NB-UVB exert their synergistic beneficial effects on psoriasis likely through regulation of MMP13 expression and secretion. Further study is necessary to reveal the detailed molecular mechanism involved in the improvement of psoriasis by retinoids and NB-UVB.
